# Supplementary material for: An external sodium ion binding site controls allosteric gating in TRPV1 channels
Source: eLife. 2016 Feb 12;5:e13356. doi: 10.7554/eLife.13356 (PMC4764576; doi:10.7554/eLife.13356)
Supplement: Figure 7—source data 1. — DOI: http://dx.doi.org/10.7554/eLife.13356.022 [file elife-13356-fig7-data1.docx]

| **Figure 7 – source data 1. Analytical expressions for P_o_ in different models.** | |
| --- | --- |
| **Model** | **P_o_** |
| (A)  Model i  (with caps.) | $U_{0}=1+J_{1}(T)+CJ_{1}\left( T \right)J_{2}\left( T \right)$  $U_{1}=K_{1}+GK_{1}J_{1}\left( T \right)+GHK_{1}J_{1}\left( T \right)J_{2}\left( T \right)$  $U_{2}=L(1+DJ_{1}\left( T \right)+DEJ_{1}\left( T \right)J_{2}\left( T \right))$  $U_{3}={L(FK}_{1}+{DFGK}_{1}J_{1}\left( T \right)+DEFGHK_{1}J_{1}\left( T \right)J_{2}\left( T \right)$)  $U_{4}=K_{2}+AK_{2}J_{1}\left( T \right)+ABK_{2}J_{1}\left( T \right)J_{2}\left( T \right)$  $U_{5}={BK}_{1}K_{2}+A{BGK}_{1}K_{2}J_{1}\left( T \right)+A{BGHK}_{1}K_{2}J_{1}\left( T \right)J_{2}\left( T \right)$  $U_{6}=L(CK_{2}+ACDK_{2}J_{1}\left( T \right)+ACDEK_{2}J_{1}\left( T \right)J_{2}\left( T \right))$  $U_{7}={L(BCFK}_{1}K_{2}+ABCDFGK_{1}K_{2}J_{1}\left( T \right)+A{BCDEFGHK}_{1}K_{2}J_{1}\left( T \right)J_{2}\left( T \right))$  $P_{o}=\frac{U_{2}+U_{3}+U_{6}+U_{7}}{U_{0}+U_{1}+U_{2}+U_{3}+U_{4}+U_{5}+U_{6}+U_{7}}$ |
| (B)  Model ii | $P_{o}=\frac{J_{1}\left( T \right)L+J_{1}\left( T \right)J_{2}\left( T \right)L+GK_{1}J_{1}\left( T \right)J_{3}\left( T \right)}{1+J_{1}\left( T \right)+J_{1}\left( T \right)L+J_{1}\left( T \right)J_{2}\left( T \right)L+K_{1}+GK_{1}J_{1}\left( T \right)+GK_{1}J_{1}\left( T \right)J_{3}\left( T \right)}$ |
| (C)  Model iii | $U_{0}=1+J_{1}\left( T \right)$  $U_{1}=K_{1}+GK_{1}J_{1}\left( T \right)$  $U_{2}=L(1+DJ_{1}\left( T \right))$  $U_{3}={L(FK}_{1}+{DFGK}_{1}J_{1}\left( T \right)$)  $P_{o}=\frac{U_{2}+U_{3}}{U_{0}+U_{1}+U_{2}+U_{3}}$ |
| U_x_ are auxiliary functions used to group different states in the model.  $J\left( T \right)=\exp\left( -\frac{\Delta H^{o}-T\Delta S^{o}}{RT} \right)$; For Fig. 7-Supplement 6: $J\left( T \right)=exp(\frac{\Delta S^{o}\left( T_{0} \right)}{R}-\frac{\Delta C_{p}}{R}\left( 1-\frac{T_{0}}{T}+\ln\left( \frac{T_{0}}{T} \right) \right))$  K_1_ = (K_1_’ x [Na^+^])^1.5^; K_2_ = (K_2_’ x [Capsaicin])^1.5^  L, K_1_’, K_2_’, A, B, C, D, E, F, G, H are constants. | |
