# Supplementary material for: An external sodium ion binding site controls allosteric gating in TRPV1 channels
Source: eLife. 2016 Feb 12;5:e13356. doi: 10.7554/eLife.13356 (PMC4764576; doi:10.7554/eLife.13356)
Supplement: Figure 7—source data 2. — DOI: http://dx.doi.org/10.7554/eLife.13356.023 [file elife-13356-fig7-data2.docx]

| **Figure 7 – source data 2. Parameters for the mathematical gating models.** | | | | | |
| --- | --- | --- | --- | --- | --- |
|  | **(A)**  **model i** | **(B)**  **model ii** | **(C)**  **model iii** | **(D)^b^**  **model i** | **(E)^c^**  **model i** |
| ΔH^o^_1_  (kcal/mol) | 30 | 30 | 20 | / | 30 |
| ΔS^o^_1_  (kcal mol^-1^ K^-1^) | 0.112 | 0.1125 | 0.073 | / | 0.112 |
| ΔH^o^_2_  (kcal/mol) | 50 | 40 | / | / | 50 |
| ΔS^o^_2_  (kcal mol^-1^ K^-1^) | 0.158 | 0.119 | / | / | 0.158 |
| ΔH^o^_3_  (kcal/mol) | / | 50 | / | / | / |
| ΔS^o^_3_  (kcal mol^-1^ K^-1^) | / | 0.163 | / | / | / |
| L | 0.04 | 0.27 | 2 x 10^-5^ | 0.04 | 0.04 |
| K_1_’ (M^-1^) | 2 x 10^3^ | 3 x 10^3^ | 1.2 x 10^3^ | 2 x 10^3^ | **20 x 10^3^** |
| K_2_’ (M^-1^) | 30 x 10^3^ | / | ^/^ | / | 30 x 10^3^ |
| A | 0.8 | / | / | / | 0.8 |
| A’^a^ | 1 | / | / | 1 | **0.1** |
| B | 300 | / | / | / | **0.05** |
| C | 35 x 10^3^ | / | 32 x 10^3^ | / | 35 x 10^3^ |
| D | 6.5 | / | 12 x 10^3^ | 6.5 | 6.5 |
| E | 10 | / | / | 10 | 10 |
| F | 2 x 10^-3^ | / | 10 | 2 x 10^-3^ | 2 x 10^-3^ |
| G | 15 x 10^-3^ | 0.01 | / | 15 x 10^-3^ | 15 x 10^-3^ |
| H | 10 x 10^-3^ | / | / | 10 x 10^-3^ | 10 x 10^-3^ |
| ΔS^o^_1_(T_0_)  (kcal mol^-1^ K^-1^) | / | / | / | -0.011 | / |
| ΔC_p,1_  (kcal mol^-1^ K^-1^) | / | / | / | 0.4 | / |
| T_0-1_ (K) | / | / | / | 210 | / |
| ΔS^o^_2_(T_0_)  (kcal mol^-1^ K^-1^) | / | / | / | -0.048 | / |
| ΔC_p,2_  (kcal mol^-1^ K^-1^) | / | / | / | 0.55 | / |
| T_0-2_ (K) | / | / | / | 200 | / |
| ^a^ A’ is determines the coupling between capsaicin binding and J_2_ in model i.  ^b^ Parameters in (D) are for model i with a change in heat capacity (see Methods) associated with J_1_ and J_2_.  ^c^ Parameters in (E) for model i and apply to TRPV1 Δ604-626 (see Fig. 9 – Supplement 1A). The numbers in bold are those that changed relative to the parameters for simulating WT TRPV1 channels. | | | | | |
